# Multilocus Sequence Typing (MLST) for Characterization of *Enterobacter cloacae*

**DOI:** 10.1371/journal.pone.0066358

**Published:** 2013-06-11

**Authors:** Tohru Miyoshi-Akiyama, Kayoko Hayakawa, Norio Ohmagari, Masahiro Shimojima, Teruo Kirikae

**Affiliations:** 1 Department of Infectious Diseases, National Center for Global Health and Medicine, Shinjuku-ku, Tokyo, Japan; 2 Disease Control and Prevention Center, National Center for Global Health and Medicine, Toyama, Shinjuku-ku, Tokyo, Japan; 3 BML Inc., Matoba, Kawagoe, Saitama, Japan; The University of Hong Kong, China

## Abstract

*Enterobacter cloacae* is an important emerging pathogen, which sometime causes respiratory infection, surgical site infection, urinary infection, sepsis, and outbreaks at neonatal units. We have developed a multilocus sequence typing (MLST) scheme utilizing seven housekeeping genes and evaluated the performance in 101 clinical isolates. The MLST scheme yielded 83 sequence types (ST) including 78 novel STs found in the clinical isolates. These findings supported the robustness of the MLST scheme developed in this study.

## Introduction

*Enterobacter cloacae* is an important emerging pathogen, which sometime causes respiratory infection, surgical site infection, urinary infection, sepsis, and outbreaks at neonatal units [1]-[4]. Extended-spectrum β-lactamases (ESBLs) and carbapenemases have been reported to be widespread in *E. cloacae* [5]. The factors dominantly contributing to drug resistance of *E. cloacae* are the plasmid-encoded CTX-M family of ESBLs, the KPC family of serine carbapenemases, and the VIM, IMP, and NDM-1 metallo-b-lactamases [5], [6]. Several molecular epidemiological methods, including pulsed-field gel electrophoresis, restriction fragment length polymorphism, and ribotyping, are routinely applied for typing of bacteria. In addition to those methods, multilocus sequence typing (MLST) is becoming a gold standard method with advances in sequencing technology. MLST can also be used to analyze the genetic relations between isolates. Therefore, MLST would be useful for analysis of the epidemiology of *E. cloacae*. Although molecular typing methods have been applied to characterize clinical isolates of *E. cloacae* [7], [8], previous studies focused mostly on discrimination of drug resistance genes. Recently, methods for discriminating *E. cloacae* complex comprised of *Enterobacter asburiae*, *E. cloacae*, *Enterobacter hormaechei*, *Enterobacter kobei*, *Enterobacter ludwigii*, and *Enterobacter nimipressuralis* based on *hsp60* and *rpoB* genotyping, multilocus sequence analysis, and comparative genomic hybridization have been evaluated [9]. MLST for *E. cloacae* has not been reported previously. Here, we designed an MLST scheme for *E. cloacae* based on seven housekeeping genes and evaluated its performance for discriminating clinical isolates.

## Materials and Methods

### Bacterial strains

Five *E. cloacae* strains the complete genome sequences of which have been determined (ATCC 13047, NCTC 9394, ENHKU 01, SCF1, and EcWSU 1; hereafter, genome strains) were used to design PCR primers. One hundred one clinical isolates collected at National Center for Global Health and Medicine Hospital and a commercial clinical laboratory (BML inc, Saitama, Japan) during 2007–2013 were used to evaluate the performance of the MLST scheme developed in the present study (Table 1).

**Table 1:** *E. cloacae* strains/clinical isolates used in this study and accession numbers of target sequences.

		Target gene							Accession # or isolation year
Strain/Isolate	ST	*dnaA*	*fusA*	*gyrB*	*leuS*	*pyrG*	*rplB*	*rpoB*	
ATCC13047	1	1	1	1	1	1	1	1	NC_014121.1
EcWSU1	2	2	2	2	2	2	2	2	NC_016514.1
ENHKU01	3	3	3	3	3	3	3	3	NC_018405.1
NCTC9394	4	4	4	4	4	4	4	4	FP929040.1
SCF1	5	5	5	2	5	5	5	5	NC_014618.1
NCGM1	6	6	6	4	6	6	4	6	2007
NCGM2	7	7	7	5	7	7	6	7	2007
NCGM3	69	7	8	5	7	8	6	7	2007
NCGM4	77	8	9	6	8	9	6	8	2011
NCGM5	74	8	33	6	9	9	6	8	2012
NCGM6	78	8	9	6	9	9	6	8	2012
NCGM7	75	8	33	7	9	9	6	8	2012
NCGM8	83	9	6	8	6	10	4	6	2012
NCGM9	82	9	6	14	10	11	4	6	2012
NCGM10	78	8	9	6	9	9	6	8	2012
NCGM11	73	8	33	6	9	12	6	8	2012
NCGM12	71	8	33	6	11	9	6	8	2012
NCGM13	74	8	33	6	9	9	6	8	2012
NCGM14	8	10	10	9	12	13	4	33	2012
NCGM15	9	11	4	4	13	14	4	9	2012
NCGM16	74	8	33	6	9	9	6	8	2012
NCGM17	78	8	9	6	9	9	6	8	2012
NCGM18	76	8	9	10	9	9	6	8	2012
NCGM19	70	8	33	11	9	9	6	8	2012
NCGM20	78	8	9	6	9	9	6	8	2012
NCGM21	78	8	9	6	9	9	6	8	2012
NCGM22	72	8	33	6	14	9	6	8	2012
NCGM23	74	8	33	6	9	9	6	8	2012
NCGM24	74	8	33	6	9	9	6	8	2012
NCGM25	55	42	11	52	37	23	16	3	2012
NCGM26	36	32	12	22	31	31	8	28	2012
NCGM27	58	44	32	12	9	35	6	6	2012
NCGM28	50	4	4	4	6	37	4	25	2012
NCGM29	39	35	25	35	47	48	12	20	2012
NCGM30	66	52	21	20	44	45	4	6	2012
NCGM31	64	50	20	17	44	45	12	32	2012
NCGM32	59	45	27	31	56	25	11	27	2012
NCGM33	62	48	4	15	42	39	4	9	2012
NCGM34	32	3	24	3	35	3	16	17	2012
NCGM35	27	26	16	25	53	22	9	15	2012
NCGM36	26	25	31	24	52	21	9	15	2012
NCGM37	30	29	18	32	33	29	8	30	2012
NCGM38	54	41	3	54	37	3	15	17	2012
NCGM39	20	19	2	46	26	51	2	13	2012
NCGM40	79	9	22	14	6	39	4	9	2012
NCGM41	67	7	34	5	7	15	6	7	2012
NCGM42	46	4	4	4	13	39	4	6	2012
NCGM43	12	13	2	45	24	52	2	14	2012
NCGM44	78	8	9	6	9	9	6	8	2012
NCGM45	28	27	14	26	54	26	10	16	2012
NCGM46	25	24	14	43	52	27	18	21	2012
NCGM47	38	34	18	33	32	30	8	31	2012
NCGM48	41	37	25	49	30	49	21	20	2012
NCGM49	17	16	2	45	25	55	7	14	2012
NCGM50	40	36	26	36	49	50	12	20	2012
NCGM51	20	19	2	46	26	51	2	13	2012
NCGM52	34	30	18	38	29	34	8	22	2012
NCGM53	43	39	27	50	48	49	12	26	2012
NCGM54	20	19	2	46	26	51	2	13	2012
NCGM55	13	13	2	45	27	56	2	14	2012
NCGM56	45	4	4	14	6	39	4	6	2012
NCGM57	78	8	9	6	9	9	6	8	2012
NCGM58	29	28	14	27	55	20	10	15	2012
NCGM59	57	43	3	51	36	18	16	19	2012
NCGM60	33	3	3	53	37	19	16	19	2012
NCGM61	63	49	20	19	45	45	4	32	2012
NCGM62	78	8	9	6	9	9	6	8	2012
NCGM63	65	51	4	21	41	42	4	6	2012
NCGM64	51	4	4	4	6	37	4	6	2012
NCGM65	18	17	13	44	19	2	2	14	2012
NCGM66	50	4	4	4	6	37	4	25	2012
NCGM67	10	11	4	4	40	39	4	6	2012
NCGM68	53	40	17	39	15	46	11	10	2012
NCGM69	11	12	2	48	18	54	13	14	2012
NCGM70	52	4	8	18	43	40	4	25	2012
NCGM71	23	22	15	39	17	47	11	10	2012
NCGM72	81	9	4	15	13	43	4	24	2012
NCGM73	78	8	9	6	9	9	6	8	2012
NCGM74	31	3	24	3	35	17	16	17	2012
NCGM76	19	18	2	41	22	51	2	13	2012
NCGM77	68	7	8	5	7	36	6	7	2012
NCGM79	21	20	30	28	50	16	20	12	2012
NCGM80	48	4	4	4	39	41	4	25	2012
NCGM81	15	14	2	30	20	51	2	14	2012
NCGM82	14	13	2	47	23	53	2	14	2012
NCGM83	47	4	4	4	39	39	19	25	2012
NCGM84	80	9	4	14	6	11	4	9	2012
NCGM85	49	4	4	4	40	38	4	23	2012
NCGM86	50	4	4	4	6	37	4	25	2012
NCGM87	78	8	9	6	9	9	6	8	2012
NCGM88	78	8	9	6	9	9	6	8	2012
NCGM89	62	48	4	15	42	39	4	9	2012
NCGM90	16	15	2	40	21	52	2	14	2012
NCGM91	50	4	4	4	6	37	4	25	2012
NCGM92	24	23	15	23	16	28	11	11	2012
NCGM94	56	42	3	52	37	23	16	3	2012
NCGM95	37	33	19	34	28	32	8	29	2012
NCGM96	35	31	19	42	31	33	17	28	2013
NCGM97	44	4	23	13	38	37	4	6	2013
NCGM98	42	38	28	37	46	49	14	20	2013
NCGM99	78	8	9	6	9	9	6	8	2013
NCGM100	24	23	15	23	16	28	11	11	2013
NCGM101	22	21	29	29	34	24	11	18	2013
NCGM102	60	46	20	19	44	45	12	6	2013
NCGM103	32	3	24	3	35	3	16	17	2013
NCGM104	61	47	8	16	51	44	6	7	2013

NCGM75, NCGM78 and NCGM93 were unused in thie study. All isolates named with NCGM were collected during 2007-2013 at laboratories located in Japan.

### Bacterial growth and biochemical identification

All strains were stored at –80°C, plated on sheep blood agar (Nissui Plate Sheep Blood Agar; Nissui, Tokyo, Japan) and cultured at 37°C overnight. Biochemical characterization was performed by Microscan Walkaway96SI (Siemens Healthcare Diagnostic. Inc., West Sacramento, CA) and VITEK 2 (SYSMEX bioMérieux Co., Ltd., Lyon, France) in a hospital laboratory and at a clinical testing company.

### DNA preparation

Bacteria were grown on sheep blood agar at 37°C overnight. A single colony was suspended in molecular biology grade water, and the suspension was heated at 95°C for 5 min. After centrifugation, the supernatant was used as the PCR template.

### Primers for MLST

The MLST scheme was developed according to the general guidelines described previously [10]. Primers to amplify internal fragments of candidate genes were designed based on the five genome strains (Table 2). Sequences of the target genes in the five strains were aligned to choose suitable region for the primers using Genetyx (Genetyx Corporation, Tokyo, Japan). Candidate genes were selected based on previously published genotyping schemes for members of the *E. cloacae* complex [9] and *dnaA* was added to increase the resolution. The primers targeted seven housekeeping genes (*dnaA*, *fusA*, *gyrB*, *leuS*, *pyrG*, *rplB*, and *rpoB*) (Table 2).

**Table 2:** Primers for *E. cloacae* MLST scheme.

	Name	Sequence (5′->3′)	Position in the target gene
Amplification primers	dnaA-f2	AYAACCCGCTGTTCCTBTATGGCGGCAC	500–527*
	dnaA-r	KGCCAGCGCCATCGCCATCTGACGCGG	1222–1248*
	fusA-f2	TCGCGTTCGTTAACAAAATGGACCGTAT	413–440*
	fusA-r2	TCGCCAGACGGCCCAGAGCCAGACCCAT	1291–1318
	gyrB-f	TCGACGAAGCGCTCGCGGGTCACTGTAA	143–170
	gyrB-r	GCAGAACCGCCCGCGGAGTCCCCTTCCA	1268–1295
	leuS-f2	GATCARCTSCCGGTKATCCTGCCGGAAG	1342–1369*
	leuS-r	ATAGCCGCAATTGCGGTATTGAAGGTCT	2159–2186*
	pyrG-f	AYCCBGAYGTBATTGCRCAYMAGGCGAT	56–83*
	pyrG-r	GCRCGRATYTCVCCCTSHTCGTCCCAGC	563–590*
	rplB-f	GTAAACCGACATCTCCGGGTCGTCGCCA	17–44*
	rplB-r	ACCTTTGGTCTGAACGCCCCACGGAGTT	735–762*
	rpoB-f	CCGAACCGTTCCGCGAACATCGCGCTGG	252–280*
	rpoB-r2	CCAGCAGATCCAGGCTCAGCTCCATGTT	973–1000*
Sequencing primers*	gyrB-r3-seq	GCAGAACCGCCCGCGGAGTCCCCTTCC	1269–1295*
	gyrB-f3-seq	AAAACCGGTACYATGGTGCGTTTCTGG	484–510*
	fusA-r2-seq	ATCTCTTCACGYTTGTTAGCGTGCATCT	1094–1121*

* These primers were used for sequencing of respective amplicons.

### PCR conditions and amplicon sequencing

The amplification reactions were performed in 20 µL using 1 µL of DNA extract as the template. The temperature program was as follows: 2 min of initial denaturation at 95°C followed by 25 cycles of denaturation at 95°C for 15 s, annealing at 50°C for 10 s, and primer extension at 72°C for 60 s. After confirmation of amplification by electrophoresis, the PCR amplicons were treated with ExoSAP-IT (USB, Cleveland, OH) to remove the excess primers according with the manufacturer's instructions, and sequenced using the primers listed in Table 2 by the dideoxy chain termination method on an ABI 3130XL Genetic analyzer or an ABI 3730XL DNA analyzer (Applied Biosystems, Foster City, CA).

### Sequence alignment and phylogenetic analysis

Genetyx (Genetyx Corporation, Tokyo, Japan) was utilized to align and edit the sequences of five *E. cloacae* genome strains as well as those obtained from the clinical isolates by Sanger sequencing. Phylogenetic analysis using concatenated MLST loci created by the STRAT2 software [11] was performed using CLUSTAL W hosted by DNA Data Bank of Japan (https://www.ddbj.nig.ac.jp). Phylogenetic tree was drawn using FigTree v1.4 (http://tree.bio.ed.ac.uk/software/figtree/). Circles indicate each clade. The START2 software was used to generate the concatenated loci sequence and calculate the number of nucleotide differences and ratio of nonsynonymous to synonymous substitutions (d*N*/d*S*) [11]. Tajima's D statistic [12], Fu's F and D statistic [13] and Ramos-Onsins & Rozas' R2 [14] were analyzed using DnaSP 5.10.1 [15].

### Index of association

To examine linkage disequilibrium among the seven genes analyzed in this study, the index of association (I_A_) values were calculated in START2 by the classical (Maynard Smith) and standardized (Haubold) methods [11].

### Accession numbers of sequences determined in this study

DNA sequences of the alleles determined in this study was deposited in DNA databank of Japan under the accession number following. The accession numbers are listed in Table 6.

## Results and Discussion

### Development of a MLST scheme for *E. cloacae*

The PCR primers designed for the *E. cloacae* MLST scheme are listed in Table 2. Candidate genes were selected based on previously published genotyping schemes for members of the *E. cloacae* complex [9] and *dnaA* was added to increase the resolution. Because *hsp60* was also included in the genotyping scheme in the previous study, we designed several combinations of primer sets and attempted to obtain amplicons. However, none of the clinical isolates tested yielded the amplicon. Thus, *hsp60* was omitted from the MLST scheme. The target amplicon sizes of *dnaA* and *gyrB* were larger than 1 kb (Table 3) to locate the primers in the conserved sequence. The percentage of variable sites at each locus ranged from 2.8 (*rplB*) to 40.9 (*pyrG*) (Table 3). The discriminatory ability of the different loci, measured as number of alleles, varied from 21 (*rplB*) to 56 (*leuS* and *pyrG*) (Table 4). The average number of alleles at each locus was 43.9, providing the potential to distinguish approximately 2.1×10^11^ different sequence types (STs). The *fusA* locus had the highest dN/dS nonsynonymous (change of amino acid) to synonymous (no change of amino acid) substitution ratio. In contrast, the dN/dS ratio of *dnaA* was close to zero, suggesting that *dnaA* is under strong selection pressure. The *rplB* gene was omitted from the genotyping scheme in the previous study [9] because of a possibility that the gene is under positive selection pressure based on the two neutrality tests: Tajima's D statistic [12] and Fu's F_s_ statistic [13]. To validate departure of neutrality of each gene, we performed additional neutrality test: Ramos-Onsins & Rozas' R_2_ test, which is more powerful at detecting population growth [14]. The R_2_ test did not detect any deviation from random evolution among any of the populations (Table 5), suggesting that it can not be excluded that *rplB* is also under neutral evolution. Thus, *rplB* was also included in the MLST scheme designed in this study. Among the 106 *E. cloacae* strains/isolates included in this study, 83 different STs were identified. Seventy-six of these STs were represented by only one strain. The data will be registered at pubmlst.org [16] to provide public analysis to MLST for *E. cloacae*. Clonality analysis of *E. cloacae* strains/isolates

**Table 3:** Characteristics of *E. cloacae* MLST loci.

Locus	*dnaA*	*fusA*	*gyrB*	*leuS*	*pyrG*	*rplB*	*rpoB*
Amplicon size (bp)	1151	906	1153	845	535	746	944
Sequence target size (bp)	442	646	434	578	259	607	545
dN/dS ratio^#^	0.0019	0.1682	0.0274	0.023	0.0576	0.0166	0.028
Number of variable sites*	71	59	60	104	106	17	77
Percentage of variable sites	16.1	9.1	13.8	18.0	40.9	2.8	14.1

* Based on the sequences of the genome strains.

# Nonsynonymous synonymous to synonymous substitution ratio.

**Table 4:** Allele frequencies of the MLST scheme for *E. cloacae.*

Allele	*dnaA*	*fusA*	*gyrB*	*leuS*	*pyrG*	*rplB*	*rpoB*
1	1	1	1	1	1	1	1
2	1	12	2	1	2	11	1
3	5	5	4	1	4	1	3
4	13	18	13	1	1	26	1
5	1	1	4	1	1	1	1
6	1	3	21	10	1	30	12
7	4	1	1	4	1	1	5
8	24	4	1	1	1	5	24
9	5	14	1	22	23	2	5
10	1	1	1	1	1	2	2
11	2	1	1	1	2	6	2
12	1	1	1	1	1	5	1
13	3	1	1	3	1	1	4
14	1	3	4	1	1	1	8
15	1	3	3	1	1	1	3
16	1	1	1	2	1	7	1
17	1	1	1	1	1	1	4
18	1	3	1	1	1	1	1
19	3	2	2	1	1	1	2
20	1	3	1	1	1	1	4
21	1	1	1	1	1	1	1
22	1	1	1	1	1	-	1
23	2	1	2	1	2	-	1
24	1	3	1	1	1	-	1
25	1	2	1	1	1	-	7
26	1	1	1	3	1	-	1
27	1	2	1	1	1	-	1
28	1	1	1	1	2	-	2
29	1	1	1	1	1	-	1
30	1	1	1	1	1	-	1
31	1	1	1	2	1	-	1
32	1	1	1	1	1	-	2
33	1	10	1	1	1	-	1
34	1	1	1	1	1	-	-
35	1	1	1	3	1	-	-
36	1	-	1	1	1	-	-
37	1	-	1	4	6	-	-
38	1	-	1	1	1	-	-
39	1	-	2	2	7	-	-
40	1	-	1	2	1	-	-
41	1	-	1	1	1	-	-
42	2	-	1	2	1	-	-
43	1	-	1	1	1	-	-
44	1	-	1	3	1	-	-
45	1	-	3	1	4	-	-
46	1	-	3	1	1	-	-
47	1	-	1	1	1	-	-
48	2	-	1	1	1	-	-
49	1	-	1	1	3	-	-
50	1	-	1	1	1	-	-
51	1	-	1	1	5	-	-
52	1	-	2	2	2	-	-
53	-	-	1	1	1	-	-
54	-	-	1	1	1	-	-
55	-	-	-	1	1	-	-
56	-	-	-	1	1	-	-
Unique	52	34	54	56	56	21	33

**Table 5:** Anlaysis of neutrality tests of genes used to develope the MLST scheme.

	Tajima's D	Fu and Li's D*	Fu and Li's F*	R2
*dnaA*	−0.51656ns	−1.10953ns	−1.05928ns	0.10537ns
*fusA*	−2.56811*	−4.52388*	−4.56688*	0.11307ns
*gyrB*	−0.75309ns	−1.08782ns	−1.14955ns	0.10381ns
*leuS*	−0.75309ns	−1.08782ns	−1.14955ns	0.10381ns
*pyrG*	−1.55553ns	−4.00283*	−3.65452*	0.10252ns
*rplB*	−2.60808*	−4.22457*	−4.36152*	0.12713ns
*rpoB*	−1.35637ns	−2.48230ns	−2.48825ns	0.11489ns

Tajima's D statistic [12], Fu's D and F statistic [13] and Ramos-Onsins & Rozas' R2 [14] were analyzed using DnaSP 5.10.1 [15].

* Statistically significant (P<0.05).

ns: Non significant.

**Figure 1:**
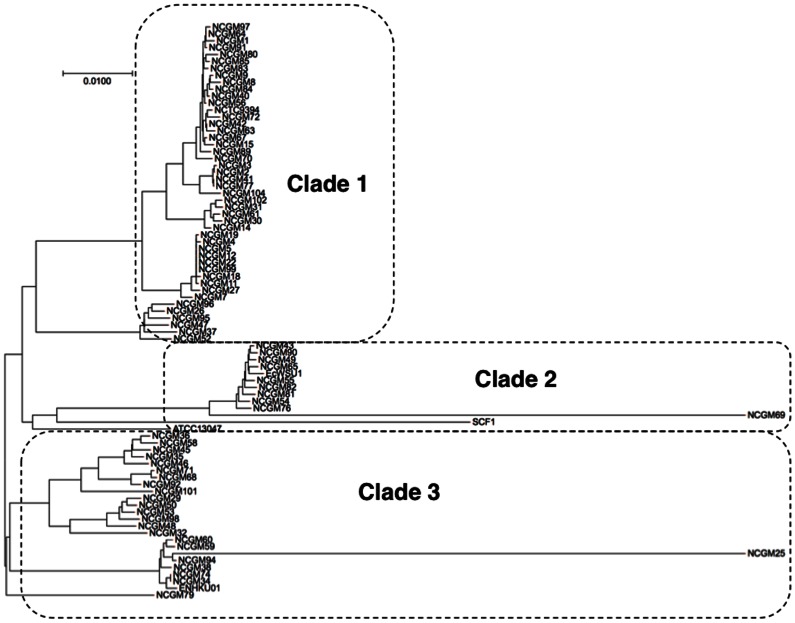
Unrooted UPGMA tree of concatenated sequences from combinations of seven MLST loci. Phylogenetic analysis using concatenated MLST loci created by the STRAT2 software was performed using CLUSTAL W hosted by DNA Data Bank of Japan (https://www.ddbj.nig.ac.jp). The dataset used contained only one isolate/ST to prevent bias toward a clonal population for strains with the same epidemiological history. The tree was drawn using FigTree v1.4 (http://tree.bio.ed.ac.uk/software/figtree/). Circles indicate each clade.

To analyze the clonality of the strains/isolates, phylogenetic analysis using the concatenated sequence consisting of the loci was performed. The dataset used contain only one isolate/ST to prevent bias toward a clonal population for strains with the same epidemiological history. These strains clustered into three clades (Figure 1). To measure the extent of linkage equilibrium within a population by quantifying the amount of recombination among a set of sequences and detecting associations between alleles at different loci, I_A_ values [17] were calculated for each clade. I_A_ values of each clade indicated significant linkage disequilibrium between alleles (clade 1:I_A_ = 0.1593, *P*<0.001; clade 2: I_A_ = 0.1857, *P*<0.001; clade 3: I_A_ = 0.3184, *P*<0.001), and thus, a clonal structure of the population studied.

In conclusion, a robust and portable typing scheme for *E. cloacae* was established. This method, based on seven housekeeping genes, separated the species into three distinct lineages. The MLST scheme developed in this study could be used for further analysis of the epidemiology of *E. cloacae*. Thus, if homologous recombination does exist, it rarely contributes to the evolution of *E. cloacae*. Sequence data analysis revealed that large number of synonymous substitutions were detected in genes *dnaA*, *gyrB*, *leuS*, *rplB* and *rpoB*, suggesting that most nonsilent mutations are eliminated through purifying selection.

**Table 6:** Accession number of allele identified in this study.

dnaA		fusA		gyrB		leuS	
Allele	Accession #	Allele	Accession #	Allele	Accession #	Allele	Accession #
dnaA_allele1	AB774293	fusA_allele1	AB774304	gyrB_allele1	AB774314	leuS_allele1	AB774325
dnaA_allele2	AB774294	fusA_allele2	AB774305	gyrB_allele2	AB774315	leuS_allele2	AB774326
dnaA_allele3	AB774295	fusA_allele3	AB774306	gyrB_allele3	AB774316	leuS_allele3	AB774327
dnaA_allele4	AB774296	fusA_allele4	AB774307	gyrB_allele4	AB774317	leuS_allele4	AB774328
dnaA_allele5	AB774297	fusA_allele5	AB774308	gyrB_allele5	AB774318	leuS_allele5	AB774329
dnaA_allele6	AB774298	fusA_allele6	AB774309	gyrB_allele6	AB774319	leuS_allele6	AB774330
dnaA_allele7	AB774299	fusA_allele7	AB774310	gyrB_allele7	AB774320	leuS_allele7	AB774331
dnaA_allele8	AB774300	fusA_allele8	AB774311	gyrB_allele8	AB774321	leuS_allele8	AB774332
dnaA_allele9	AB774301	fusA_allele9	AB774312	gyrB_allele9	AB774322	leuS_allele9	AB774333
dnaA_allele10	AB774302	fusA_allele10	AB774313	gyrB_allele10	AB774323	leuS_allele10	AB774334
dnaA_allele11	AB774303	fusA_allele11	AB809745	gyrB_allele11	AB774324	leuS_allele11	AB774335
dnaA_allele12	AB809704	fusA_allele12	AB809746	gyrB_allele12	AB809769	leuS_allele12	AB774336
dnaA_allele13	AB809705	fusA_allele13	AB809747	gyrB_allele13	AB809770	leuS_allele13	AB774337
dnaA_allele14	AB809706	fusA_allele14	AB809748	gyrB_allele14	AB809771	leuS_allele14	AB774338
dnaA_allele15	AB809707	fusA_allele15	AB809749	gyrB_allele15	AB809772	leuS_allele15	AB809812
dnaA_allele16	AB809708	fusA_allele16	AB809750	gyrB_allele16	AB809773	leuS_allele16	AB809813
dnaA_allele17	AB809709	fusA_allele17	AB809751	gyrB_allele17	AB809774	leuS_allele17	AB809814
dnaA_allele18	AB809710	fusA_allele18	AB809752	gyrB_allele18	AB809775	leuS_allele18	AB809815
dnaA_allele19	AB809711	fusA_allele19	AB809753	gyrB_allele19	AB809776	leuS_allele19	AB809816
dnaA_allele20	AB809712	fusA_allele20	AB809754	gyrB_allele20	AB809777	leuS_allele20	AB809817
dnaA_allele21	AB809713	fusA_allele21	AB809755	gyrB_allele21	AB809778	leuS_allele21	AB809818
dnaA_allele22	AB809714	fusA_allele22	AB809756	gyrB_allele22	AB809779	leuS_allele22	AB809819
dnaA_allele23	AB809715	fusA_allele23	AB809757	gyrB_allele23	AB809780	leuS_allele23	AB809820
dnaA_allele24	AB809716	fusA_allele24	AB809758	gyrB_allele24	AB809781	leuS_allele24	AB809821
dnaA_allele25	AB809717	fusA_allele25	AB809759	gyrB_allele25	AB809782	leuS_allele25	AB809822
dnaA_allele26	AB809718	fusA_allele26	AB809760	gyrB_allele26	AB809783	leuS_allele26	AB809823
dnaA_allele27	AB809719	fusA_allele27	AB809761	gyrB_allele27	AB809784	leuS_allele27	AB809824
dnaA_allele28	AB809720	fusA_allele28	AB809762	gyrB_allele28	AB809785	leuS_allele28	AB809825
dnaA_allele29	AB809721	fusA_allele29	AB809763	gyrB_allele29	AB809786	leuS_allele29	AB809826
dnaA_allele30	AB809722	fusA_allele30	AB809764	gyrB_allele30	AB809787	leuS_allele30	AB809827
dnaA_allele31	AB809723	fusA_allele31	AB809765	gyrB_allele31	AB809788	leuS_allele31	AB809828
dnaA_allele32	AB809724	fusA_allele32	AB809766	gyrB_allele32	AB809789	leuS_allele32	AB809829
dnaA_allele33	AB809725	fusA_allele33	AB809767	gyrB_allele33	AB809790	leuS_allele33	AB809830
dnaA_allele34	AB809726	fusA_allele34	AB809768	gyrB_allele34	AB809791	leuS_allele34	AB809831
dnaA_allele35	AB809727			gyrB_allele35	AB809792	leuS_allele35	AB809832
dnaA_allele36	AB809728			gyrB_allele36	AB809793	leuS_allele36	AB809833
dnaA_allele37	AB809729			gyrB_allele37	AB809794	leuS_allele37	AB809834
dnaA_allele38	AB809730			gyrB_allele38	AB809795	leuS_allele38	AB809835
dnaA_allele39	AB809731			gyrB_allele39	AB809796	leuS_allele39	AB809836
dnaA_allele40	AB809732			gyrB_allele40	AB809797	leuS_allele40	AB809837
dnaA_allele41	AB809733			gyrB_allele41	AB809798	leuS_allele41	AB809838
dnaA_allele42	AB809734			gyrB_allele42	AB809799	leuS_allele42	AB809839
dnaA_allele43	AB809735			gyrB_allele43	AB809800	leuS_allele43	AB809840
dnaA_allele44	AB809736			gyrB_allele44	AB809801	leuS_allele44	AB809841
dnaA_allele45	AB809737			gyrB_allele45	AB809802	leuS_allele45	AB809842
dnaA_allele46	AB809738			gyrB_allele46	AB809803	leuS_allele46	AB809843
dnaA_allele47	AB809739			gyrB_allele47	AB809804	leuS_allele47	AB809844
dnaA_allele48	AB809740			gyrB_allele48	AB809805	leuS_allele48	AB809845
dnaA_allele49	AB809741			gyrB_allele49	AB809806	leuS_allele49	AB809846
dnaA_allele50	AB809742			gyrB_allele50	AB809807	leuS_allele50	AB809847
dnaA_allele51	AB809743			gyrB_allele51	AB809808	leuS_allele51	AB809848
dnaA_allele52	AB809744			gyrB_allele52	AB809809	leuS_allele52	AB809849
				gyrB_allele53	AB809810	leuS_allele53	AB809850
				gyrB_allele54	AB809811	leuS_allele54	AB809851
						leuS_allele55	AB809852
						leuS_allele56	AB809853

pyrG		rplB		rpoB	
Allele	Accession #	Allele	Accession #	Allele	Accession #
pyrG_allele1	AB774339	rplB_allele1	AB774353	rpoB_allele1	AB774361
pyrG_allele2	AB774340	rplB_allele2	AB774354	rpoB_allele2	AB774362
pyrG_allele3	AB774341	rplB_allele3	AB774355	rpoB_allele3	AB774363
pyrG_allele4	AB774342	rplB_allele4	AB774356	rpoB_allele4	AB774364
pyrG_allele5	AB774343	rplB_allele5	AB774357	rpoB_allele5	AB774365
pyrG_allele6	AB774344	rplB_allele6	AB809896	rpoB_allele6	AB774366
pyrG_allele7	AB774345	rplB_allele7	AB809897	rpoB_allele7	AB809912
pyrG_allele8	AB774346	rplB_allele8	AB809898	rpoB_allele8	AB809913
pyrG_allele9	AB774347	rplB_allele9	AB809899	rpoB_allele9	AB809914
pyrG_allele10	AB774348	rplB_allele10	AB809900	rpoB_allele10	AB809915
pyrG_allele11	AB774349	rplB_allele11	AB809901	rpoB_allele11	AB809916
pyrG_allele12	AB774350	rplB_allele12	AB809902	rpoB_allele12	AB809917
pyrG_allele13	AB774351	rplB_allele13	AB809903	rpoB_allele13	AB809918
pyrG_allele14	AB774352	rplB_allele14	AB809904	rpoB_allele14	AB809919
pyrG_allele15	AB809854	rplB_allele15	AB809905	rpoB_allele15	AB809920
pyrG_allele16	AB809855	rplB_allele16	AB809906	rpoB_allele16	AB809921
pyrG_allele17	AB809856	rplB_allele17	AB809907	rpoB_allele17	AB809922
pyrG_allele18	AB809857	rplB_allele18	AB809908	rpoB_allele18	AB809923
pyrG_allele19	AB809858	rplB_allele19	AB809909	rpoB_allele19	AB809924
pyrG_allele20	AB809859	rplB_allele20	AB809910	rpoB_allele20	AB809925
pyrG_allele21	AB809860	rplB_allele21	AB809911	rpoB_allele21	AB809926
pyrG_allele22	AB809861			rpoB_allele22	AB809927
pyrG_allele23	AB809862			rpoB_allele23	AB809928
pyrG_allele24	AB809863			rpoB_allele24	AB809929
pyrG_allele25	AB809864			rpoB_allele25	AB809930
pyrG_allele26	AB809865			rpoB_allele26	AB809931
pyrG_allele27	AB809866			rpoB_allele27	AB809932
pyrG_allele28	AB809867			rpoB_allele28	AB809933
pyrG_allele29	AB809868			rpoB_allele29	AB809934
pyrG_allele30	AB809869			rpoB_allele30	AB809935
pyrG_allele31	AB809870			rpoB_allele31	AB809936
pyrG_allele32	AB809871			rpoB_allele32	AB809937
pyrG_allele33	AB809872			rpoB_allele33	AB809938
pyrG_allele34	AB809873				
pyrG_allele35	AB809874				
pyrG_allele36	AB809875				
pyrG_allele37	AB809876				
pyrG_allele38	AB809877				
pyrG_allele39	AB809878				
pyrG_allele40	AB809879				
pyrG_allele41	AB809880				
pyrG_allele42	AB809881				
pyrG_allele43	AB809882				
pyrG_allele44	AB809883				
pyrG_allele45	AB809884				
pyrG_allele46	AB809885				
pyrG_allele47	AB809886				
pyrG_allele48	AB809887				
pyrG_allele49	AB809888				
pyrG_allele50	AB809889				
pyrG_allele51	AB809890				
pyrG_allele52	AB809891				
pyrG_allele53	AB809892				
pyrG_allele54	AB809893				
pyrG_allele55	AB809894				
pyrG_allele56	AB809895				
